# Supplementary material for: End Sequence Analysis Toolkit (ESAT) expands the extractable information from single-cell RNA-seq data
Source: Genome Res. 2016 Oct;26(10):1397–410. doi: 10.1101/gr.207902.116 (PMC5052061; doi:10.1101/gr.207902.116)
Supplement: Supplemental Material [file supp_gr.207902.116_Supp_Materials.tar.gz › ESAT-esat_v0.1/doc/html/classbroad_1_1core_1_1siphy_1_1_evolutionary_model_parameters-members.html]

ESAT: Member List


ESAT


- Main Page
- Related Pages
- Packages
- Classes
- Files

- Class List
- Class Index
- Class Hierarchy
- Class Members

All Classes Namespaces Files Functions Variables Enumerator Pages

- broad
- core
- siphy
- EvolutionaryModelParameters

broad.core.siphy.EvolutionaryModelParameters Member List

This is the complete list of members for broad.core.siphy.EvolutionaryModelParameters, including all inherited members.

|  |  |  |
| --- | --- | --- |
| copy() | broad.core.siphy.EvolutionaryModelParameters |  |
| EvolutionaryModelParameters(Phylogeny tree, double kappa, double mu, Matrix Q, Matrix pi) | broad.core.siphy.EvolutionaryModelParameters |  |
| EvolutionaryModelParameters(File sourceFile) | broad.core.siphy.EvolutionaryModelParameters |  |
| EvolutionaryModelParameters(double kappa, double[] bg, double mu) | broad.core.siphy.EvolutionaryModelParameters |  |
| generateHKYQMatrix(double kappa, double scalingFactor) | broad.core.siphy.EvolutionaryModelParameters |  |
| getBackgroundNucleotideFreqs() | broad.core.siphy.EvolutionaryModelParameters |  |
| getRateMatrix() | broad.core.siphy.EvolutionaryModelParameters |  |
| getTree() | broad.core.siphy.EvolutionaryModelParameters |  |
| setBackgroundNucleotideFreqs(Matrix dist) | broad.core.siphy.EvolutionaryModelParameters |  |
| setRateMatrix(Matrix Q) | broad.core.siphy.EvolutionaryModelParameters |  |
| setTree(Phylogeny tree) | broad.core.siphy.EvolutionaryModelParameters |  |
| toString() | broad.core.siphy.EvolutionaryModelParameters |  |
| write(BufferedWriter bw) | broad.core.siphy.EvolutionaryModelParameters |  |


---

Generated on Fri Aug 15 2014 15:10:57 for ESAT by  

 1.8.7
